# The Use of a Biopolymer Conjugate for an Eco-Friendly One-Pot Synthesis of Palladium-Platinum Alloys

**DOI:** 10.3390/polym11121948

**Published:** 2019-11-27

**Authors:** Daniele Silvestri, Stanisław Wacławek, Rohith K. Ramakrishnan, Abhilash Venkateshaiah, Kamil Krawczyk, Vinod V. T. Padil, Bartłomiej Sobel, Miroslav Černík

**Affiliations:** 1 Institute for Nanomaterials, Advanced Technologies and Innovation, Technical University of Liberec, 46117 Liberec, Czech Republic; daniele.silvestri@tul.cz (D.S.); rohith.kunjiparambil.ramakrishnan@tul.cz (R.K.R.); abhilash.venkateshaiah@tul.cz (A.V.); kamil.krawczyk@tul.cz (K.K.); vinod.padil@tul.cz (V.V.T.P.); 2 Institute of Engineering Materials and Biomaterials, Faculty of Mechanical Engineering, Silesian University of Technology, 44–100 Gliwice, Poland; bartlomiej.sobel@gmail.com

**Keywords:** green synthesis, biopolymers, bimetallic nanoparticles, catalytic reduction, 4-nitrophenol

## Abstract

Raising health and environmental concerns over the nanoparticles synthesized from hazardous chemicals have urged researchers to focus on safer, environmentally friendlier and cheaper alternatives as well as prompted the development of green synthesis. Apart from many advantages, green synthesis is often not selective enough (among other issues) to create shape-specific nanoparticle structures. Herein, we have used a biopolymer conjugate and Pd and Pt precursors to prepare sustainable bimetallic nanoparticles with various morphology types. The nanoparticles were synthesized by a novel green approach using a bio-conjugate of chitosan and polyhydroxybutyrate (Cs-PHB). The bio-conjugate plays the simultaneous roles of a reducing and a capping agent, which was confirmed by attenuated total reflection Fourier transform infrared spectroscopy (ATR-FTIR) and energy dispersive X-ray spectrometry (EDS) analysis, proving the presence of a Cs-PHB layer on the surface of the prepared nanoparticles. The EDS profile also revealed the elemental structure of these nanoparticles and confirmed the formation of a Pd/Pt alloy. TEM morphological analysis showed the formation of star-like, octahedron or decahedron Pd/Pt nanoparticles, depending on the synthesis conditions. The bimetallic Pd/Pt nanoparticles synthesized with various Pd/Pt molar ratios were successfully applied for the catalytic reduction of 4-nitrophenol to 4-aminophenol by borohydride. The calculated κc values (ratio of *k*_app_ to the concentration of the catalyst) revealed that the decahedron nanoparticles (size of 15 ± 4 nm), synthesized at the molar ratio of 2:1 (Pd/Pt), temperature of 130 °C, 10 g/L of Cs-PHB conjugate and time of 30 min, exhibited excellent catalytic activity compared to other bimetallic nanoparticles reported in the literature.

## 1. Introduction

The raising health and environmental concerns over nanoparticles synthesized from hazardous chemicals, which are also often economically unfeasible, have urged researchers to focus on safer, environmentally-friendlier and cheaper alternatives. These reasons have prompted the development of green nanoparticle syntheses, which are safe and adhere to the green chemistry approach [1]. Biopolymers, which are abundantly available and easily biodegradable, are promising materials for providing an environmentally-benign synthesis of nanomaterials. These natural polymers have been successfully used as reducing, stabilizing and capping agents in the synthesis of nanoparticles [2,3,4], allowing alterations in the nanoparticle size [5] and shape [6]. Furthermore, the different functional groups present in these biopolymers can actively contribute to the improvement of metallic nanoparticle catalytic reactions [7,8]. A typical example of a simple monometallic and bimetallic nanoparticle synthesis is the one-step reduction and stabilization of Au and Ag nanoparticles [9,10]. 

Chitosan is considered one of the most studied biopolymers in the literature, and has been successfully used in different applications such as the synthesis and stabilization of various different nanoparticles [11,12,13], bio-medical applications [14], drug delivery [15], water treatment [16] and many other uses [17,18,19]. Chitosan is obtained from chitin, which is mainly extracted from crustacean shell wastes [20]. While its non-toxicity for mammals and biodegradability make it popular, its insolubility in water is one of its drawbacks.

Another biopolymer that is starting to gain interest in different scientific fields is poly(3-hydroxybutyrate) (PHB), which can be produced by different bacteria [21] and also from waste materials [22,23]. It is usually used as a carbon source in *in-situ* bioremediation [24], a drug delivery carrier [25], a biodegradable bioplastic [26], and a stabilizing agent for nanoparticles [27]. However, difficulties such as the solubility of PHB in only organic solvents, which are toxic to both humans and the environment [28,29], need to be addressed to achieve good dispersions for synthesis.

Motivated by the above situation, our group developed a water-soluble conjugate of chitosan and PHB, which was successfully applied to control not only the growth and aggregation of the Au nanoparticles but also their surface properties [30].

Due to interactions between two metals and their unique and more flexible surface structures in comparison to monometallic nanoparticles, bimetallic nanoparticles have gained precedence over traditional heterogeneous catalysts due to their excellent catalytic activity [31]. The nanoparticle surface area plays a key role in heterogeneous catalysis because it is directly correlated to the catalyst active sites on which the catalytic reactions are taking place. Moreover, the nanoparticles are often easily recovered from the reaction medium, and they possess steric environments within their active sites, both features that can positively influence the catalytic activity [32]. Among the noble metals, both palladium (Pd) and platinum (Pt) are well known for their unique characteristics, and both are used successfully in different scientific fields, including catalysis [33,34,35,36,37,38]. Due to the fact they have similar face-centred cubic (fcc) crystal structures and a high lattice match (lattice mismatch of 0.77%), palladium and platinum are highly miscible [39,40]. 

We propose a one-pot, quick and green synthesis of decahedral Pd/Pt using solely Pd and Pt precursors and a Cs-PHB bio-conjugate as a reducing reagent. Based on our previous studies, we hypothesize that Cs-PHB cannot only help to control the growth and aggregation of the nanoparticles but also to tailor their catalytic activity. To the best of our knowledge, this is the first report to use Cs-PHB for the green synthesis of bimetallic nanoparticles. In addition, we believe in the simplicity of this procedure for obtaining decahedron Pd/Pt bimetallic nanoparticles. The synthesized nanoparticles were characterized by ATR-FTIR, TEM, and EDS, and successfully tested on the standard reduction reaction of the 4-nitrophenol (4-NP) to 4-aminophenol (4-AP).

## 2. Materials and Methods 

### 2.1. Reagents and Solutions

Chitosan (low *M*_w_ of 50–190 kDa, 75%–85% deacetylated), sodium borohydride (98%), 4‑nitrophenol (ReagentPlus, >99%), K_2_PdCl_4_ (98%), PtCl_4_ (96%) were purchased from Sigma–Aldrich (Saint Louis, MO, USA); polyhydroxybutyrate (PHB, Biomer P209) from Biomer (Krailing, Germany); nitric acid (65%) from Lach-ner (Neratovice, Czech Republic). Deionized water (DI; 18.2 MΩ·cm^–1^, ELGA, Veolia Water, Marlow, UK) was used in all of the experiments. 

### 2.2. Analytical Methods

ATR-FTIR spectra were recorded at a resolution of 4 cm^−1^ over the 4000–700 cm^−1^ range using a NICOLET IZ10 spectrometer (Thermo Scientific, Waltham, MA, USA) equipped with a germanium ATR crystal and a single reflection angle 45° horizontal ATR accessory. The UV-Vis spectroscopic analysis was performed using a DR 3900 UV-Vis spectrophotometer (Hach Lange, Loveland, CO, USA) equipped with 1 cm quartz cuvettes. High-resolution transmission electron microscopy (HR-TEM) analysis was carried out using transmission electron microscopy/scanning transmission electron microscopy (TEM/STEM) system (Titan 80-300, FEI, city, state abbrev if USA, country) with a super twin-lens operated at 300 kV and equipped with an annular dark field detector. The presence of various elements in the obtained nanoparticles was analysed using energy-dispersive X-ray spectroscopy (EDX, Aztec, Oxford Instruments, Abingdon, UK). ICP-MS (Elan 6000, Perkin Elmer, Waltham, MA, USA) was used to determine the Pd/Pt concentration.

### 2.3. Preparation of Cs-PHB Conjugate

The conjugate was prepared following the procedure reported previously by our group [30]. Briefly, a chitosan solution was made by adding chitosan (0.5 g) to acidified deionized water (100 mL) and stirring to obtain a homogeneous solution. Subsequently, PHB (0.12 g) was added to the mixture and stirred overnight at 80 °C. The resulting solution was sonicated for 30 min at 80 °C, purified by a dialysis tube, and finally freeze-dried.

### 2.4. Synthesis of Bimetallic Nanoparticles

Pd/Pt bimetallic nanoparticles were synthesized following a modified co-reduction method of Lim et al. [41]. Briefly, K_2_PdCl_4_ and PtCl_4_ were dissolved in DI to get two (Pd and Pt) stock solutions with a concentration of 10 mM each. Both solutions were stirred for 5 minutes in order to dissolve the salts. Cs-PHB was dissolved in DI to get a stock solution of 20 g/L. Subsequently, a certain amount of palladium and platinum precursor stock solutions (0.5 mL of Pd and 0.5 mL of Pt precursor stock solution for the Pd:Pt ratio of 1:1; 0.25 mL of Pd and 0.5 mL of Pt precursor stock solution for the Pd:Pt ratio of 1:2; 0.5 mL of Pd and 0.25 mL of Pt precursor stock solution for the Pd:Pt ratio 2:1) were added to the Cs-PHB solution (2.5 mL), the volume was raised to 5 mL by adding DI. The reactor was heated (130–150 °C) for 30 min following the procedure reported by Venkateshaiah et al. [42]. The reaction was stopped by cooling down the samples in cold water. The obtained nanoparticles were washed three times with deionized water and stored in a refrigerator (4 °C) for future use. 

### 2.5. Catalytic Test

The catalytic test of 4-NP reduction to 4-AP by NaBH_4_ was carried out in a standard 1 cm path length quartz cuvette. The procedure was reported previously by Baruah et al. [43]. A typical test involves the mixing of 24 µL of 4-NP (5 mM), and an excess of NaBH_4_ (120 µL of 0.1 M) in an Eppendorf tube (1.5 mL). A certain amount of nanoparticles was added, and the volume was adjusted to 1 mL using DI water. Then the solution was immediately transferred into a quartz cuvette and the absorbance was recorded by UV-Vis at regular intervals. All of the tests were carried out at room temperature (25 °C) in triplicate. An excess of NaBH_4_ (12 mM of NaBH_4_ and 0.12 mM of 4-NP) was used in the reduction process.

## 3. Results and Discussion

Pd/Pt nanoparticles were synthesized under different conditions (temperature from 130 to 150 °C) and using different ratios of Pd and Pt precursors (from 1:2 to 2:1). The resulting particles were characterised by ATR-FTIR, HR-TEM and EDS. Three types of nanoparticles synthetized at a constant temperature but at different metallic ratio were also compared for their catalytic activity. 

### 3.1. Characterization of the Nanoparticles

#### 3.1.1. ATR-FTIR

**Figure 1:**
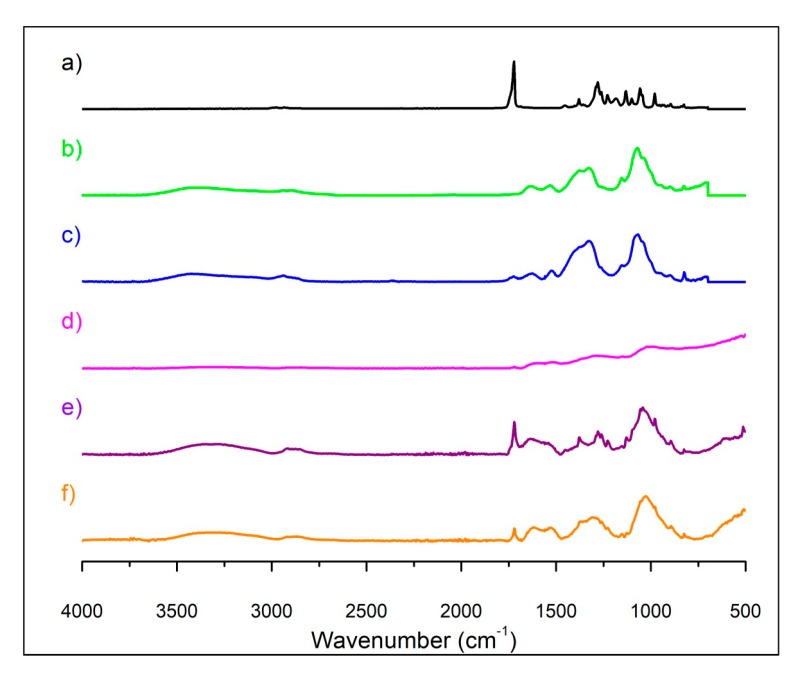
ATR-FTIR analysis of (**a**) PHB, (**b**) chitosan, (**c**) Cs-PHB, (**d**) Pd/Pt ratio 1:1 (zoom on the region of the 4000–1500 cm^−1^ spectrum part is available in Figure S1 in Supplementary Materials), (**e**) Pd/Pt ratio 1:2 and (**f**) Pd/Pt ratio 2:1 (synthesis temperature of Pd/Pt: 130 °C).

An ATR-FTIR analysis was performed to examine the functional groups located on the Pd/Pt bimetallic nanoparticles (Figure 1). The peak observable at ~1724 cm^−1^ in the PHB spectrum (Figure 1a) may be attributed to the ester group present in the PHB. The chitosan spectrum shows a peak at ~3300 cm^−1^ due to the O–H and N–H bonds, whereas the peak at ~2900 cm^−1^ may be ascribed to the symmetric or asymmetric CH_2_ stretching vibrations. The peak at ~1600 cm^−1^ may be assigned to the NH_2_ groups, while at ~1380 cm^−1^ the peak may be ascribed to CH_3_ symmetrical deformations [44]. The last representative peak at ~1100 cm^−1^ may be attributed to C–O–C glycosidic linkage. The conjugate spectrum (Figure 1c) shows differences when comparing the PHB (Figure 1a) to the chitosan (Figure 1b) spectra. A decrease in the intensity of the NH_2_ group at ~1600 cm^−1^ [30] was observed, while an increase in the intensity at ~1555 cm^−1^ was observed, which may correspond to the amide type II bond formation [30]. This suggests that the amino group of chitosan reacts with the C–O–C group of PHB to form the amide bond.

The ATR-FTIR spectra of Pd/Pt nanoparticles (Figure 1d–f) showed several bands. The first one (~3330 cm^−1^) may be attributed to the NH/OH bond, whereas the one at ~2926 cm^−1^ is compatible with asymmetric or symmetric CH_2_ stretching vibration. The peak at 1724 cm^−1^ may be related to the ester group, while the one at ~1555 cm^−1^ to the amide type II bond. The hydroxyl groups present in the conjugate may assist in the reduction of the precursor as reported by Dang et al. [45] and by Dorjnamjin et al. [46], while the rest of the polymer may coat the nanoparticles. The differences in intensity between the variously synthesized Pd/Pt nanoparticles may indicate different amounts of organic and inorganic material that could be found in samples. For example, Pd/Pt = 1:1 nanoparticles were synthesized with the highest ratio of metal precursors concentration (2 mM overall) to the polymer conjugate concentration, and in the low frequencies (<1500 cm^−1^) their spectrum exhibits a (high absorbance) baseline sloping down to the left (typical for some metal nanoparticles; similar phenomena could be observed e.g. in the work of Hu et al. [47]).

#### 3.1.2. HR-TEM

**Figure 2:**
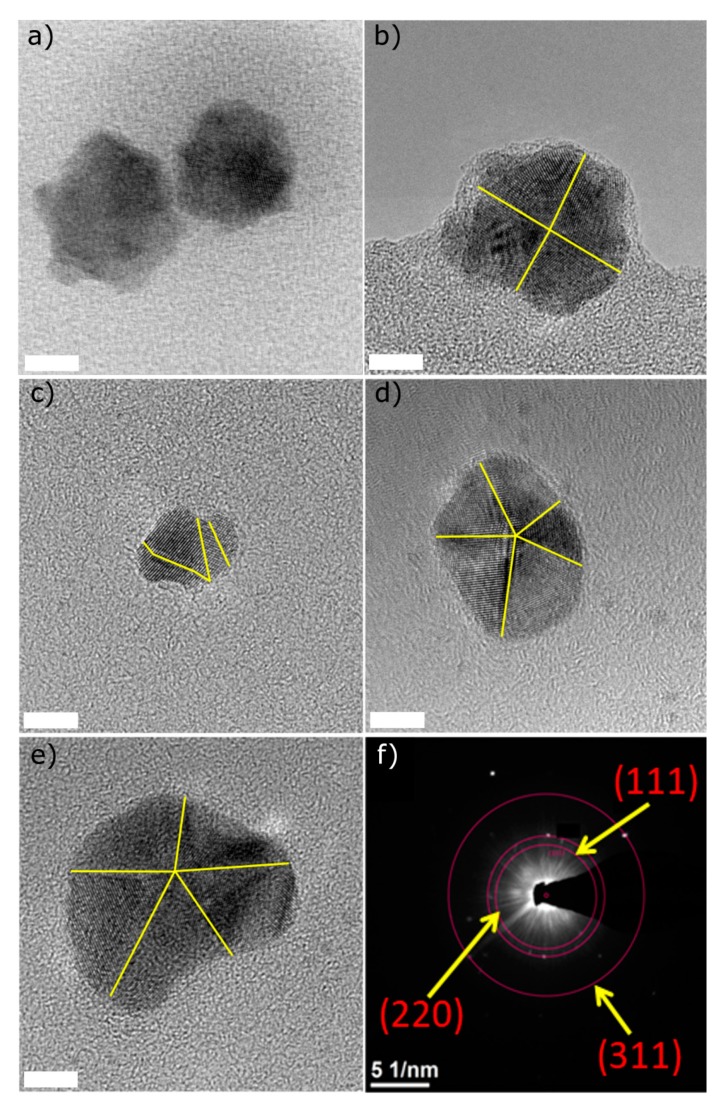
HR-TEM images of characteristic Pd/Pt nanoparticles synthesized with different molar ratios and temperatures (**a**) Pd/Pt 1:1 at 150 °C, (**b**) 1:1 at 140 °C, (**c**) 1:1 at 130 °C, and (**d**) 2:1 at 130 °C; (**e**) 1:2 at 130 °C and (**f**) SAED pattern of Pd/Pt (2:1). For all of the samples, the scale bar stands for 5 nm.

In order to obtain more information about the morphology of the synthesized Pd/Pt nanoparticles, a HR‑TEM analysis was performed. Figure 2 shows the different shapes obtained by altering the synthesis temperature (from 130 to 150 °C) and Pd/Pt molar ratio (1:1, 1:2 and 2:1).

The sample synthesized at 150 °C and with a molar ratio 1:1 (Pd/Pt) (Figure 2a) shows a star-like structure, while lowering the temperature of synthesis to 140 °C (Figure 2b) changed the morphology of the nanoparticles to an octahedron. An additional decrease of temperature to 130 °C (Figure 2c) caused a synthesis of smaller nanoparticles with a surface characterized by different, randomly-orientated faces. Moreover, when the molar ratio changed to 2:1 and 1:2 (Pd/Pt) at the remaining temperature (130 °C), nanoparticles with decahedral morphology were observed (Figure 2d,e). The decahedral shape of the nanoparticles occurs only under strict conditions [48]. When certain conditions are applied, the ions specifically interact to form a Cs-PHB/precursor complex, which determines the formation of decahedron shapes upon reduction. However, when the conditions and the ratios vary, other shapes are formed. Zhang et al. [49] reported that hydroxyl groups may affect the shape of the nanoparticles. Ghosh et al. [50] showed the possibility of obtaining flower-shaped zero-valent iron by controlling the amount of hydroxyl groups during the synthesis process, which suggests that the presence of hydroxyl groups may influence the formation of decahedral morphology. The SAED pattern for a Pd/Pt ratio of 2:1 indicates the polycrystalline nature of an as-synthesized Pd/Pt bimetallic alloy (Figure 2f). The SAED analysis identified (111), (220) and (311) planes of fcc. For this sample, the nanoparticle size distribution was calculated from TEM micrographs, and the mean size of these nanoparticles was found to be 15 ± 4 nm (Figure S2 in Supplementary Materials).

The various synthesis strategies used to obtain bimetallic Pd/Pt nanoparticles with varying morphologies are shown in Table 1. As stated earlier, changes to the synthesis procedure may result in the formation of structurally different nanoparticles, e.g. nanocubes are obtained by reduction with poly(vinylpyrrolidone) (PVP) while nanotetrahedra are formed when Na_2_C_2_O_4_ and formaldehyde are used [39]. Conventionally for these kind of reactions, high temperatures [51] and prolonged synthesis times [52,53] are required. Very often the reducing agents used are hazardous, e.g. sodium borohydride [44,45]. 

Wang et al. [59] reported the possibility to synthesize decahedral Pd/Pt, wherein the synthesis procedure can be divided into two steps: the first is to obtain Pd decahedraln structures, followed by the platinum deposition. Nano star-shaped Pd/Pt particles were reported by Lim et al. [41] using a co-reduction method involving Na_2_PdCl_4_ and K_2_PtCl_4_ in a PVP aqueous medium at 80 °C for 18 h. Another example of shape-specific synthesis of Pd/Pt was a seeded growth method using palladium truncated octahedral seeds for the synthesis of Pd/Pt nanodendrites [60]. 

#### 3.1.3. EDS, Mapping and Profile 

**Figure 3:**
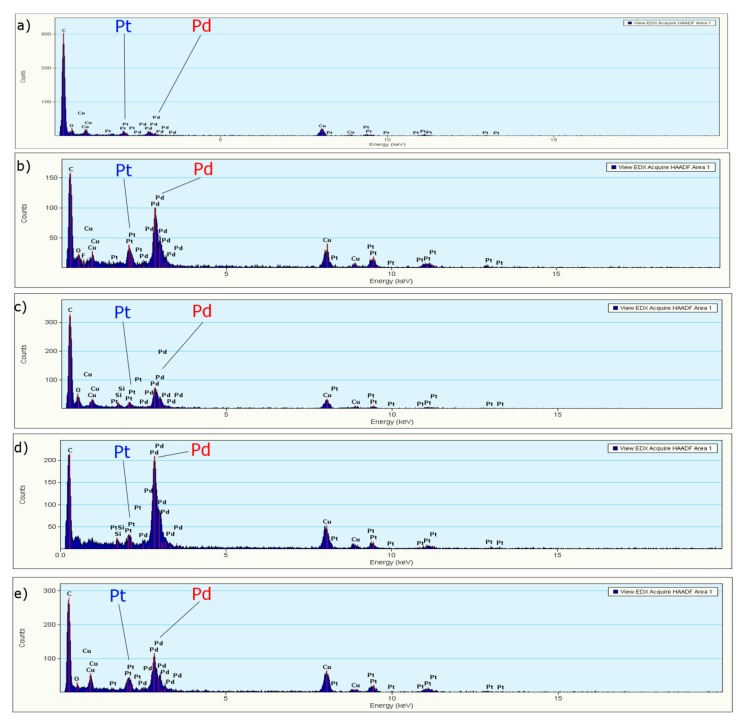
EDS analysis of Pd/Pt nanoparticles synthesized at different temperatures and molar ratios (**a**) 150 °C and 1:1, (**b**) 140 °C and 1:1, (**c**) 130 °C and 1:1, (**d**) 130 °C and 2:1, and (**e**) 130 °C and 1:2.

The EDS analysis of the bimetallic nanoparticles shows that all of the samples contain both palladium and platinum metals (Figure 3). Moreover, carbon and oxygen were also present in all of the analysed samples. The presence of both C and O may be attributed to the existence of a conjugate on the surface of these nanoparticles, which may act as a stabilizing agent. 

**Figure 4:**
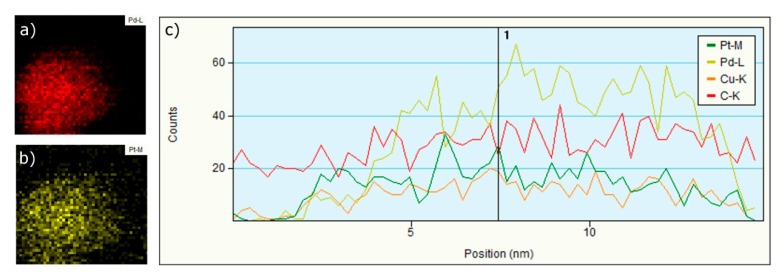
EDS mapping of (**a**) Pd and (**b**) Pt, and (**c**) EDS profile analysis of Pd/Pt nanoparticles (synthesis condition: Pd/Pt ratio 2:1, 10 g/L of Cs-PHB and temperature of 130 °C).

The EDS mapping analysis of the bimetallic nanoparticles (molar ratio of Pd/Pt of 2:1 and temperature of 130 °C) clearly shows the presence of both metals ubiquitously on the surface of the nanoparticle (Figure 4a,b). The EDS mapping also determined the presence of Pd/Pt alloy. The EDS profile analysis (Figure 4c) shows the presence of carbon (due to the presence of the conjugate), and it is in accordance with the previous ATR-FTIR analysis (see above). The profile also confirmed the predominant presence of Pd in almost all of the particle regions due to the initial Pd/Pt ratio of 2:1. 

### 3.2. Catalysis 

The catalytic performance of the Pd/Pt nanoparticles was proven by employing the reduction of 4-NP to 4-AP by NaBH_4_ as a model [61]. The aqueous 4-NP solution shows a maximum absorption at ~317 nm, which upon addition of sodium borohydride shifts to 401 nm, indicating the formation of 4-nitrophenolate, and the solution turns from pale yellow to bright yellow. The reduction does not take place in the absence of a catalyst (kinetic barrier), which was verified by the unchanged intensity of the maximum absorption at 401 nm in the absence of the catalysts for 40 min (data not shown). 

When the Pd/Pt nanoparticles were added to the solution, the intensity at 401 nm gradually decreased until it disappeared. Because an excess of NaBH_4_ was used, the pseudo first-order kinetics model was applied to evaluate the catalytic performance of the Pd/Pt nanoparticles [62]. Due to the fact that the absorbance at 401 nm was linearly dependent on the 4-NP concentration (through 4-nitrophenolate), the rate constant *k* of the reaction can be calculated from the linear plot of ln(*A*_t_/*A*_0_) versus the reaction time *t*(min) [63,64,65,66,67]:

ln(A_t_/A_0_) = −*k*_app_*t* (1)

where *A*_t_ and *A*_0_ is absorbance at time *t* and 0, respectively. The pseudo-first-order kinetic rate constants (*k*_app_) of the 4-NP reduction calculated based on Equation (1) for the various concentrations of nanoparticles synthesized with different molar (Pd/Pt) ratios of 1:1, 1:2 and 2:1, respectively are summarized in Table 2. 

**Figure 5:**
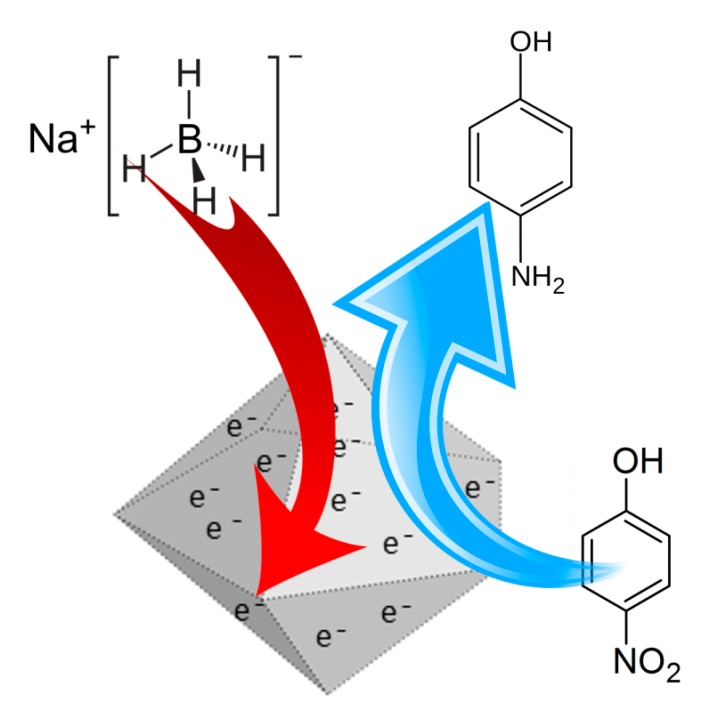
Electron transfer mechanism for reduction of 4-NP to 4-AP.

The reduction of 4-NP to 4-AP with borohydride catalysed by Pd/Pt nanoparticles may be explained by an electrochemical reaction, where the nanoparticles facilitate the electron transfer from BH_4_^−^ to 4-NP. The mechanism is divided into the following steps: first both borohydride and 4-NP are adsorbed on the surface of the Pd/Pt nanoparticles, then electrons are transferred from BH_4_^−^ to the nanoparticles with the formation of a negatively charged layer on their surface, later the electrons are transferred to 4-NP with a consequent formation of reduced products (Figure 5).

4-NP is often used for testing the catalytic activities of nanoparticles; nevertheless, catalytic performance comparisons are not easy. Most studies report only the *k*_app_, but the *k*_app_ is strongly dependent on the concentration of reactants and the catalyst used for the reaction. Increasing the amount of catalyst in the reactor increases the total surface area available for the reaction, which means that a higher reaction rate is facilitated, and the time needed for reduction is shortened. Also, it was not possible in our study to add the same amounts of catalysts at different Pd/Pt ratios during the experiments. To overcome this, the activity parameter (κ_c_) was employed to compare the efficiencies. This was determined by calculation of the slope of *k*_app_ (s^−1^) as a function of the catalyst concentration (g/L) [65].

**Figure 6:**
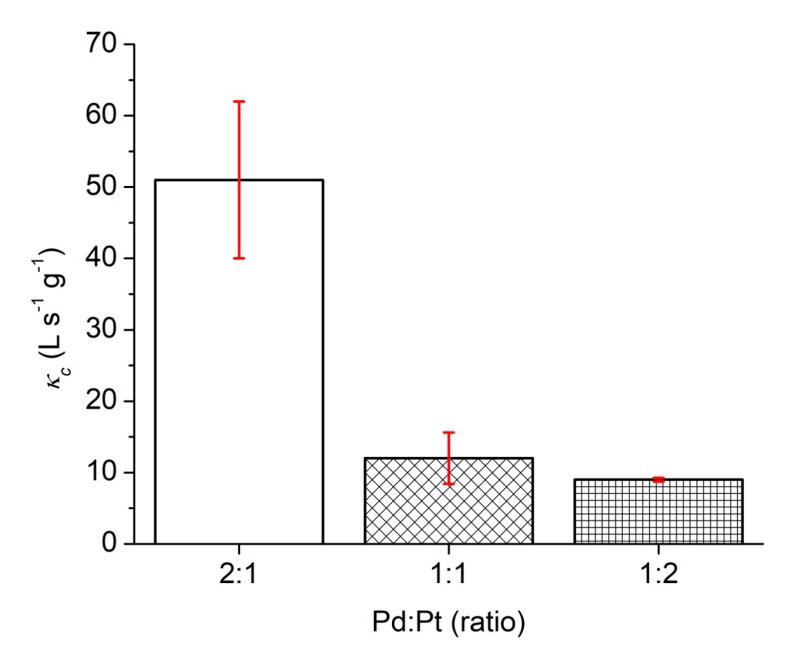
Comparison between the κ_c_ values of nanoparticles obtained by different ratios of Pd and Pt precursors (the red error bars represent the slope error).

To the best of our knowledge, κ_c_ is the most appropriate parameter for comparing the catalytic activity of catalysts reported in the literature [65,67]. The κ_c_ values were calculated for these three different Pd/Pt ratios and, therefore, different morphologies. While for the same Pd/Pt ratio (1:1) and excess of Pt (1:2), the κ_c_ value is approximately 10, for the excess of Pd (2:1), the κ_c_ value of 51 is significantly higher. This is probably caused by a higher Pd ratio and not by morphology, since both excesses of one of the metals (1:2 and 2:1) have the same morphology of a decahedron, but significantly different κ_c_ values (Figure 6).

Table 3 shows a comparison of the κ_c_ values obtained by different bimetallic catalysts for the reduction of 4-NP. The value determined by this study for a Pd/Pt ratio of 2:1 is one of the highest known to-date.

## 4. Conclusions

The present research describes a facile mediated green synthesis of bimetallic Pd/Pt nanoparticles of various morphologies. The nanoparticles were synthesized from K_2_PdCl_4_ and PtCl_4_ precursor salts by a co-reduction with a Cs-PHB conjugate. Depending on the temperature and metal ratio, nanoparticles with a star-like structure, and octahedral or decahedral morphology were formed. The optimal conditions to obtain a decahedral shape were found to be: (Pd/Pt) molar ratio of 2:1, synthesis temperature of 130 °C, 10 g/L of Cs-PHB conjugate and time of 30 min. While ATR-FTIR and EDS confirmed the presence of a Cs-PHB layer on the surface of the nanoparticles, EDS verified the formation of a Pd/Pt alloy. TEM analysis confirmed the different shapes and sizes of the nanoparticles by changing the temperature and molar metal ratio. The decahedral bimetallic nanoparticles prepared at a molar ratio of 2:1 show an excellent catalytic performance for the catalytic reduction of 4-NP by borohydride. The pseudo-first-order kinetic constant for the nanoparticles synthesized with molar ratios (Pd/Pt) of 1:1, 1:2 and 2:1 were found to be 0.898 min^−1^ (catalyst concentration: 1.515 mg/L), 0.305 min^−1^ (0.586 mg/L) and 1.968 min^−1^ (0.809 mg/L)_,_ respectively. Compared with the other bimetallic nanoparticles, the (2:1) decahedral Pd/Pt nanoparticles exhibit excellent catalytic performance, which is demonstrated by the high κ_c_ value (51 ± 11 L s^−1^ g^−1^).

## Figures and Tables

**Table 1:** Synthesis procedures reported in the literature for obtaining Pd/Pt nanoparticles with different shapes.

Shape	Solvent	Precursors	Molar Pd/Pt Ratio	Reducing Agent	Temperature (°C)	Synthesis Time (min)	Reference
Cube	DMF	Na_2_PdCl_4_ K_2_PtCl_6_	1:1	-	130	300	[54]
Nanosponges	Water	H_2_PdCl_4_ K_2_PtCl_6_	1:1	NaBH_4_	Room temperature	~5	[55]
Tetrahedron	Water	Na_2_PdCl_4_ K_2_PtCl_6_	1:1	HCHO	180	120	[56]
Octahedron	Water	Na_2_PdCl_4_ H_2_PtCl_6_	1:1	Glycerol	100	180	[57]
Corallite-like structure	Water	K_2_PdCl_4_ K_2_Pt(CN)_4_	2.05:1	NaBH_4_	Room temperature	120	[58]
Branched Dandelion-like	Water	Na_2_PdCl_4_ K_2_PtCl_6_	1:7	Ascorbic acid	Room temperature	30	[55]
Nanocages	Water	K_2_PdBr_4_ Na_2_PtBr_6_	1:2	Ascorbic acid	Room temperature	480	[40]
Irregular polyhedron	Water	K_2_PdCl_4_ PtCl_4_	1:1	Cs-PHB	130	30	This work
Decahedron	Water	K_2_PdCl_4_ PtCl_4_	1:2	Cs-PHB	130	30	This work
Decahedron	Water	K_2_PdCl_4_ PtCl_4_	2:1	Cs-PHB	130	30	This work

**Table 2:** The pseudo-first-order kinetic rate constants (*k*_app_) of Pd/Pt synthesized in different ratios and κ_c_ value obtained by linear approximation of *k*_app_ (s^−1^) vs concentration of catalysts (g/L).

Catalysts	Synthesis Temperature (°C)	Concentration (mg/L)	*k*_app_ (min^−1^)	κ_c_ (L s^−1^ g^−1^)
Pd/Pt (1:1)	130	0.379	0.038	12 ± 4
		0.757	0.546	
		1.515	0.897	
Pd/Pt (1:2)	130	0.147	0.066	9 ± 1
		0.293	0.152	
		0.586	0.305	
Pd/Pt (2:1)	130	0.202	0.198	51 ± 11
		0.404	0.424	
		0.809	1.967	

**Table 3:** Comparison of different bimetallic catalysts on the reduction of 4-NP reported in the literature.

Catalysts	Catalyst Concentration (mg/L)	4-NP Concentration (mM)	NaBH_4_ Concentration (mM)	*k*_app_ (s^−1^)	κ_c_ (L s^−1^ g^−1^)	Ref.
Pd/Au	8	0.07	21	0.258	32	[68]
Au_53_Pd_47_/graphene nanosheets	0.06	0.05	5	0.014	240	[69]
Cu/Ag	0.48	0.096	11.2	0.0003	7.18	[70]
PdCuY	20	0.72	1.5	0.002	0.12	[71]
Pd/Pt nanotubes	3.4	0.09	100	0.008	25	[72]
Pd/Pt (2:1)	0.809	0.12	12	0.033	51 ± 11	This work
Pd/Pt (1:1)	0.757	0.12	12	0.009	12 ± 4	This work
Pd/Pt (1:2)	0.586	0.12	12	0.005	9 ± 1	This work

## Supplementary Materials

The following are available online at https://www.mdpi.com/2073-4360/11/12/1948/s1, Figure S1 FTIR analysis of Pd/Pt ratio 1:1, Figure S2: Size distribution of Pd/Pt synthesized at 130 °C and molar ratio 2:1 (Pd:Pt), Figure S3: pseudo-first-order kinetics of sample synthesized with molar ratio of 1:1 with differet concentration of nanoparticles (a) 0.379 mg/L (R^2^ 0.993), (b) 0.757 mg/L (R^2^ 0.992), (c) 1.515 mg/L (R^2^ 0.982) and (d) HRTEM image of Pd/Pt decahedron nanoparticle (molar ratio 1:1 (Pd/Pt) 130 °C), Figure S4: pseudo-first-order kinetics of sample synthesized with molar ratio of 1:2 with differet concentration of nanoparticles (a) 0.147 mg/L (R^2^ 0.992), (b) 0.293 mg/L (R^2^ 0.998), (c) 0.586 mg/L (R^2^ 0.984) and (d) HRTEM image of Pd/Pt decahedron nanoparticle (molar ratio 1:2 (Pd/Pt) 130 °C), Figure S5: pseudo-first-order kinetics of sample synthesized with molar ratio of 2:1 with different concentration of nanoparticles (a) 0.202 mg/L (R^2^ 0.997) (b) 0.404 mg/L (R^2^ 0.986) (c) 0.809 g/L (R^2^ 0.979) and d) HRTEM image of Pd/Pt decahedron nanoparticle (molar ratio 2:1 (Pd/Pt) 130 °C).
