# Supplementary material for: The Use of a Biopolymer Conjugate for an Eco-Friendly One-Pot Synthesis of Palladium-Platinum Alloys
Source: Polymers (Basel). 2019 Nov 27;11(12):1948. doi: 10.3390/polym11121948 (PMC6960498; doi:10.3390/polym11121948)
Supplement: Supplementary file 1 [file polymers-11-01948-s001.pdf]

polymers

The use of a biopolymer conjugate for an eco-friendly one-pot synthesis of palladium-platinum alloys

Daniele Silvestri[1], Stanisław Wacławek[1]*, Rohith K. Ramakrishnan[1], Abhilash Venkateshaiah[1], Kamil Krawczyk[1], Vinod V.T. Padil[1], Bartłomiej Sobel[2] and Miroslav Černík[1]*

[1]Institute for Nanomaterials, Advanced Technologies and Innovation, Technical University of Liberec, , 46117 Liberec, Czech Republic

[2]Institute of Engineering Materials and Biomaterials, Faculty of Mechanical Engineering, Silesian University of Technology, 44–100 Gliwice, Poland

*Correspondence: stanislaw.waclawek@tul.cz (S.W.); miroslav.cernik@tul.cz (M.Č.)

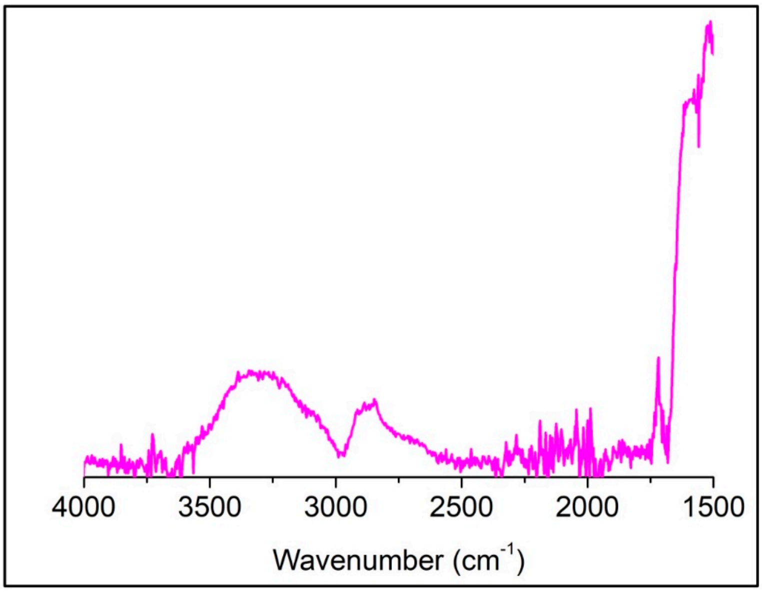

Figure 1. ATR-FTIR analysis of Pd/Pt ratio 1:1.

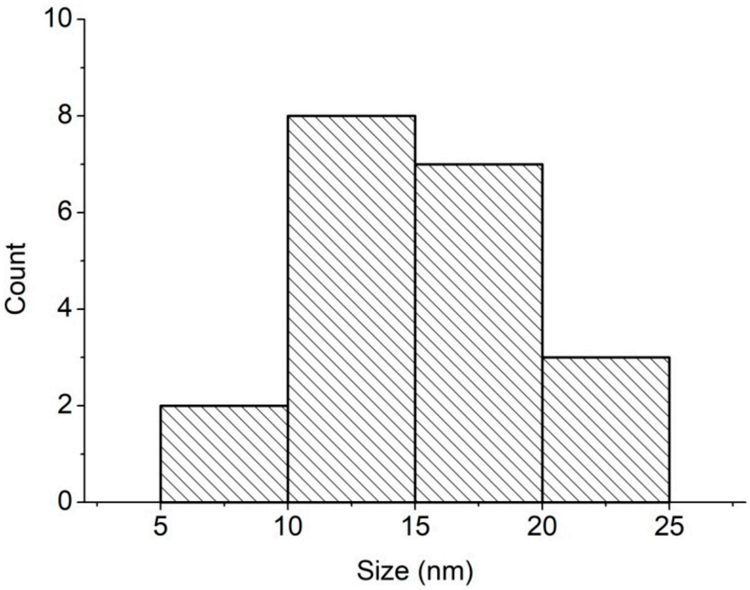

Figure 2. Size distribution of Pd/Pt synthesized at 130 °C and molar ratio 2:1 (Pd:Pt).

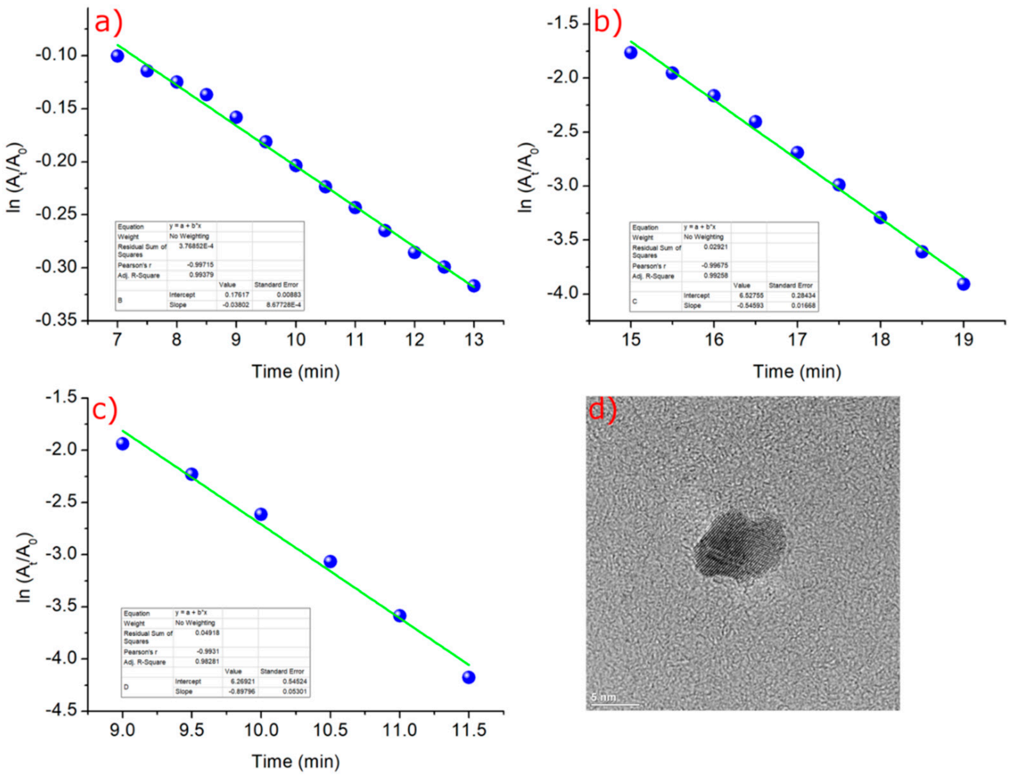

Figure 3. Pseudo-first-order kinetics of sample synthesized with molar ratio of 1:1 with differet concentration of nanoparticles (a) 0.379 mg/L (R^2 0.993), (b) 0.757 mg/L (R^2 0.992), (c) 1.515 mg/L (R^2 0.982) and (d) HRTEM image of Pd/Pt decahedron nanoparticle (molar ratio 1:1 (Pd/Pt) 130 °C).

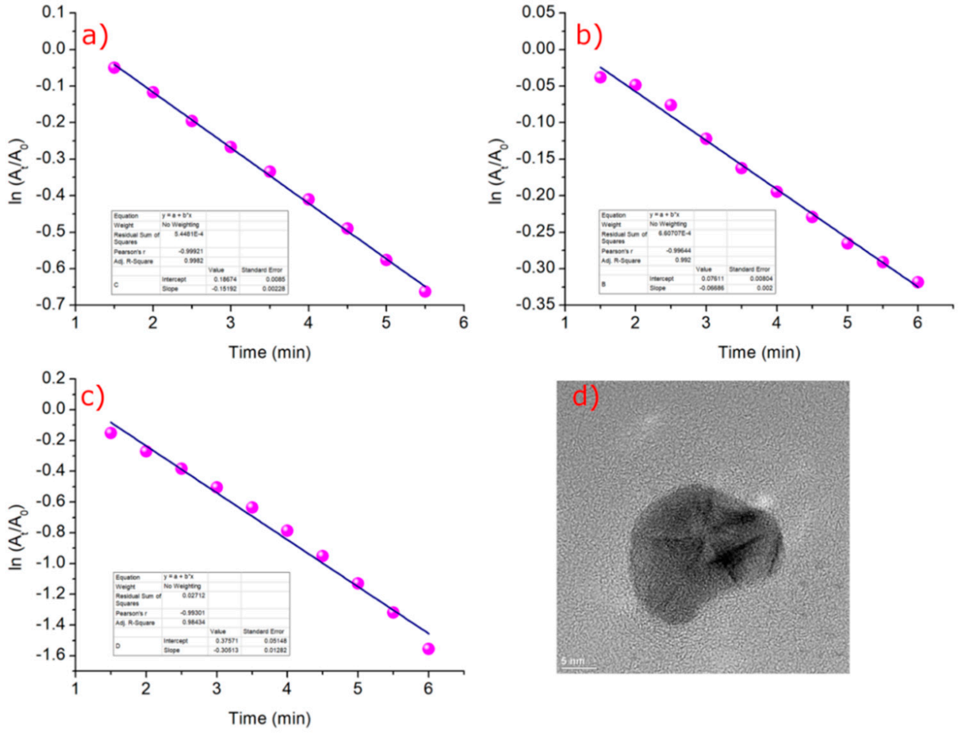

Figure 4. Pseudo-first-order kinetics of sample synthesized with molar ratio of 1:2 with differet concentration of nanoparticles (a) 0.147 mg/L (R^2 0.992), (b) 0.293 mg/L (R^2 0.998), (c) 0.586 mg/L (R^2 0.984) and (d) HRTEM image of Pd/Pt decahedron nanoparticle (molar ratio 1:2 (Pd/Pt) 130 °C).

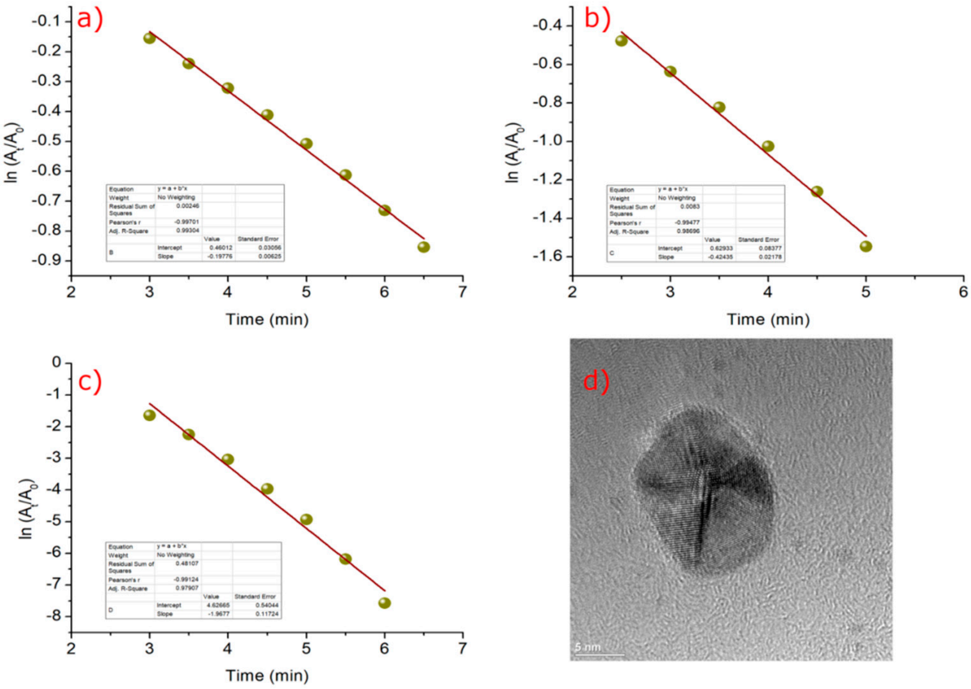

Figure 5. Pseudo-first-order kinetics of sample synthesized with molar ratio of 2:1 with differet concentration of nanoparticles (a) 0.202 mg/L (R^2 0.997) (b) 0.404 mg/L (R^2 0.986) (c) 0.809 g/L (R^2 0.979) and (d) HRTEM image of Pd/Pt decahedron nanoparticle (molar ratio 2:1 (Pd/Pt) 130 °C).
